# Exploring the effects of COVID-19 on family planning: results from a qualitative study in rural Uganda following COVID-19 lockdown

**DOI:** 10.1186/s12978-023-01566-3

**Published:** 2023-02-09

**Authors:** Katelyn M. Sileo, Christine Muhumuza, Teddy Helal, Allison Olfers, Haruna Lule, Samuel Sekamatte, Trace S. Kershaw, Rhoda K. Wanyenze, Susan M. Kiene

**Affiliations:** 1 Department of Public Health, University of Texas at San Antonio, San Antonio, TX USA; 2 Makerere University School of Public Health, Kampala, Uganda; 3 Gombe Hospital, Gombe, Uganda; 4 Yale University, New Haven, CT USA; 5 San Diego State University, San Diego, CA USA

**Keywords:** COVID-19, Family planning services, Uganda, Qualitative

## Abstract

**Background:**

The COVID-19 pandemic has likely affected the already high unmet need for family planning in low- and middle-income countries. This qualitative study used Andersen’s Behavioral Model of Health Service Use as a theoretical framework to explore the possible ways in which the COVID-19 pandemic, including the impact of a 3-month government mandated lockdown, might affect family planning outcomes in rural Uganda. A secondary aim was to elicit recommendations to improve family planning service delivery in the context of COVID-19.

**Methods:**

Between June and October 2020, we conducted four focus group discussions with men and women separately (N = 26) who had an unmet need for family planning, and 15 key-informant interviews with community leaders and family planning stakeholders. Data were analyzed using thematic analysis.

**Results:**

We identified a significant disruption to the delivery of family planning services due to COVID-19, with potential negative effects on contraceptive use and risk for unintended pregnancy. COVID-19 had a negative effect on individual enabling factors such as family income, affecting service access, and on community enabling factors, such as transportation barriers and the disruption of community-based family planning delivery through village health teams and mobile clinics. Participants felt COVID-19 lockdown restrictions exacerbated existing contextual predisposing factors related to poverty and gender inequity, such as intimate partner violence and power inequities that diminish women’s ability to refuse sex with their husband and their autonomy to use contraceptives. Recommendations to improve family planning service delivery in the context of COVID-19 centered on emergency preparedness, strengthening community health systems, and creating new ways to safely deliver contractive methods directly to communities during future COVID-19 lockdowns.

**Conclusions:**

This study highlights the consequences of COVID-19 lockdown on family planning distribution, as well as the exacerbation of gender inequities that limit women’s autonomy in pregnancy prevention measures. To improve family planning service uptake in the context of COVID-19, there is a need to strengthen emergency preparedness and response, utilize community structures for contraceptive delivery, and address the underlying gender inequities that affect care seeking and service utilization.

## Plain language summary

This study explored the potential impact of the COVID-19 pandemic and a 3-month government mandated lockdown on barriers to accessing family planning services in rural Uganda, and recommendations to improve service delivery in the event of future COVID-19 restrictions. Data were collected from four focus group discussions with men and women separately (N = 26) who had an unmet need for family planning, and 15 interviews with community leaders and family planning stakeholders. The delivery of family planning services was disrupted due to COVID-19, negatively affecting community members’ ability to access services, such as by reducing their income. COVID-19 also disrupted community and health system distribution of services, such as through a transportation ban and the suspension of all community-based family planning delivery through village health teams and mobile clinics. Participants felt that COVID-19 lockdown restrictions worsened intimate partner violence, and with men at home more, limited women’s ability to use contraceptives without their partner’s knowledge and resulted in more sex between partners without women being able to refuse. Taken together, these consequences were thought to increase women’s risk of unintended pregnancy. Recommendations to improve family planning service delivery in the context of COVID-19 centered on measures to improve the health system’s response to emergencies and to safely deliver contraceptive methods directly to communities during future COVID-19 lockdowns. The successful implementation of community-based family planning will depend on efforts to increase men’s acceptance of family planning, while addressing underlying gender inequities that diminish women’s ability to time and space pregnancy.

## Introduction

Early in the COVID-19 pandemic, it was projected that the pandemic would result in a 10% reduction in contraceptive use and a significant rise in unintended pregnancies in low and middle-income countries (LMICs) [1]. The shutdown of nonessential services, physical distancing, mandated lockdowns, and economic downturns are among the pandemic’s externalities that were expected to, and have, affected women’s reproductive health outcomes globally [1]. As of March 2021, the United Nations Population Fund (UNFPA) estimated that 12 million women in LMICs have faced barriers in accessing family planning services due to COVID-19, resulting in 1.4 million unintended pregnancies [2].

LMICs such as Uganda are particularly vulnerable to COVID-19 disruptions that affect reproductive health; Uganda has an already high fertility rate (5.45 children per woman), low contraceptive use (29.2%), and a high maternal mortality rate (375 deaths per 100,000 live births) [3–5]. Moreover, the provision of family planning and other health services during crises is challenged by the already heavily burdened healthcare systems in LMICs. As of October 18, 2021, Uganda reported a total of 125,261 COVID-19 cases and 3,185 COVID-19 deaths [6]. While the burden of COVID-19 has been less severe in sub-Saharan African countries compared to other regions of the world to-date [6], COVID-19 has had surges in several African countries including Uganda [6] and vaccine access and uptake has been slow [7, 8]. In addition, the country has undergone strict government-instituted “lockdowns” which may affect access to health services. These lockdowns include a three-month lock-down from March to June 2020 and a second 42-day lockdown starting in June 2021, lifted to a partial lockdown on July 31, 2021 [8]. Restrictions put in place during lockdown to slow COVID-19 transmission included the closure of schools and businesses, strict stay-at-home orders, and a ban on public transportation.

While it is expected that such restrictions have affected reproductive healthcare access globally [1, 9–11], research is needed that examines the ways in which the COVID-19 pandemic has affected access to and use of contraceptives in rural areas of LMICs. A few quantitative studies exist with this aim [12–16], one of which found 20% of surveyed community members in Burkina Faso, Ethiopia, and Nigeria reported difficulty in accessing family planning services due to COVID-19 [13]. To our knowledge, limited studies have qualitatively explored the possible effects of COVID-19 on family planning service delivery and access to-date [17]. In addition, researchers posit that other known determinants of family planning related to gender inequity may be exacerbated by COVID-19 [18], which may be particularly relevant in settings such as Uganda. Male partner control of family planning and intimate partner violence (IPV) are known barriers to contraceptive use [19–21], which may be intensified by lockdown orders and the social consequences of COVID-19, such as economic stress [22].

### Theoretical framework and literature review

This qualitative study uses Andersen’s Behavioral Model of Health Service Use (ABM) as a theoretical framework to explore the effect of the COVID-19 pandemic on barriers to family planning service delivery and use in rural Uganda immediately following a 3-month government mandated lockdown [23–25]. The ABM is a multilevel model used extensively to explain health service utilization [26], including contraceptive use and family planning service uptake in Uganda [20]. The model focuses on person characteristics (predisposing, enabling, and need factors), influenced by the external environment, that combine to influence health behavior (i.e., personal health practices and health service use), which influence health status outcomes (i.e., perceived and evaluated health status). Within each factor (predisposing, enabling, and need), the model differentiate between individual and contextual determinants of health service utilization [27].

Predisposing factors include individual and contextual factors that lead to a predisposition of people to use services. Examples of individual factors associated with greater contraceptive use in Uganda and other LMICs include demographic and social characteristics, such as higher education levels and being in a committed/marital relationship [28, 29]. Individual predisposing factors can also include health beliefs, i.e., attitudes, values, and knowledge related to a health behavior [27]. In Uganda, low levels of knowledge about contraceptives, misinformation about side effects, and negative attitudes, especially among men, are reported health beliefs that undermine contraceptive use [30, 31].

Contextual factors that predispose individuals to use health services include the demographic and social composition of communities, collective and organizational values, cultural norms and political perspectives [26, 27]. Fertility norms that reinforce early marriage and lower age of sexual debut are examples of contextual predisposing factors that negatively influence contraceptive use and family planning in LMICs [32]. Such norms are prevalent in rural Uganda, as are other norms reinforcing gender inequity that impede contraceptive use through male control over family planning decision-making, poor spousal communication, and IPV [20, 30, 33, 34]. Researchers posit that gender inequities and gender-based violence may be exacerbated as a result of COVID-19 stay-at-home orders, consequently hindering continued contraceptive use [18, 35].

Enabling factors are those that enable or impede use of health services. Individual enabling factors include an individual’s income, access to health insurance to pay for health services, and the price of health care. In Uganda, greater wealth is associated with greater contraceptive use [33, 36]. COVID-19’s disruptions to the economy and an individual’s ability to earn could negatively affect their ability to access services. Individual enabling factors such as means of transportation are also known barriers to health service access in rural areas of Uganda [37–39] and are likely impacted by COVID-19.

At the contextual level, community enabling factors include health system barriers, like waiting time for health care, quality of care, and the financial and organizational resources available within the community for health services, such as the rate of health insurance coverage, the relative price of goods and services, the distribution of health facilities and personnel, provider availability, and outreach and education programs [26, 27]. Research demonstrates a direct effect of COVID-19 on organizational enabling factors for health services in sub-Saharan Africa, resulting in disruptions to immunization efforts, malnutrition supplementation, family planning, and antenatal care [13].

Perceived need for health services at the individual level is defined as how people view and experience their own general health, functional state and illness symptoms. In contrast, evaluated need is an objective measurement or professional assessment of one’s health status. The ABM also defines need at the contextual level, including environmental need characteristics or environmental health conditions (e.g., occupational, traffic, or crime related injury) and population health indices (e.g., epidemiological indicators of mortality, morbidity, disability). Since the current study is a qualitative inquiry with community members, we focus on individual-level perceived need.

**Fig. 1:**
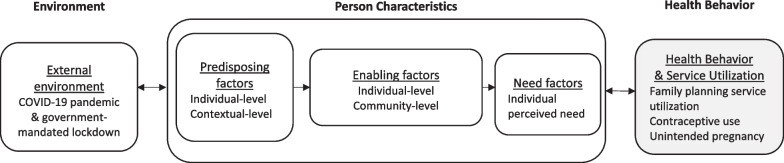
The Anderson Behavioral Model of Health Service Utilization applied to the exploration of COVID-19’s effect on barriers and facilitators to family planning service utilization

Taken together, the ABM provides an appropriate framework to explore COVID-19’s effect on family planning barriers through the perspectives of community members and family planning stakeholders who have experienced or witnessed the effects first-hand. As displayed in Fig. 1, we use the ABM to explore the interaction between COVID-19 and its societal consequences (e.g., lockdown), with predisposing, enabling, and need factors on health behavior outcomes of contraceptive access, use, and unintended pregnancy. The outcomes are not measured directly in this qualitative study, but are included in the figure to demonstrate the full conceptual framework. A secondary aim of our study is to elicit community recommendations to improve the provision of family planning amid a COVID-19 outbreak. The findings of this study can help inform the development of innovations in family planning service delivery in the context of infectious disease outbreaks and similar humanitarian crises.

## Methods

This qualitative study aimed to understand the determinants of contraceptive use in a rural area of Butambala district in central Uganda as part of the formative phase of a pilot intervention. Given the onset of the COVID-19 pandemic during the study, we expanded the aims of the study to include the exploration of the impact of COVID-19 on barriers and facilitators to contraceptive use and factors influencing risk of unintended pregnancy, which is the focus of the current paper. Data were collected from June and October 2020 in a rural district of Butambala in central Uganda immediately following a 3-month government-mandated lockdown from March to June 2020. Restrictions during lockdown included stay-at-home orders and a curfew, a ban on public transportation, the closure of nonessential businesses and schools, and social distancing measures such as the ban of group gatherings. The study was carried out with support from the Butambala District Health Team, comprised of technical health officials responsible for the strategic planning and allocation of resources to meet the health needs of the community.

Participants were recruited from three villages within the same rural district located approximately two hours from the capital city of Kampala. The population of each selected village is approximately 100 households each. All villages are largely Muslim and Christian, with polygamy commonly practiced. A governmental health center III (HCIII) located in the sub-county served the three villages, offering free contraceptives and individual and couples family planning counseling. HCIIIs offer condoms, oral pills, injectable contraceptives, intrauterine devices (IUDs), and implants. However, the contraceptives offered can vary across HCIIIs based on stock availability and the capacity of providers to offer long-acting reversible methods (LARCs). A General Hospital (HCV) in the district provides all the short-term reversible methods, LARCs, and non-reversible methods (vasectomy, tubal ligation). In addition, the Village Health Team (VHT), a cadre of community health workers, serve as a liaison between the community and health facilities, and support community family planning efforts. VHTs distribute short-term methods directly in the community (i.e., condoms, oral contraceptive pills) and provide individual and group community education about family planning. In addition, an international nongovernmental organization, Marie Stopes International, works in the district to provide regular community outreach to distribute contraceptives in selected villages within the district, including the provision of LARCs and non-reversible methods.

Methods used for data collection included focus group discussions (FGDs) and key informant interviews (KIIs). We conducted four FGDs with men and women (N = 26) recruited directly from one of the three villages with the support of a VHT. Groups were separated by age and gender to ensure participants could speak openly (women < 30 = 6; women > 30 = 7; men < 30 = 7; men > 30 = 6). Inclusion criteria included being from the selected communities, being of reproductive age (women: 18–40, or an emancipated minor, defined as individuals below 18 years who are married, have a child, or are self-sufficient; men: 18–50 or an emancipated minor), considering oneself married, speaks Luganda, not currently pregnant, and having an unmet need for family planning. An unmet need for family planning was defined as wanting to delay pregnancy for at least a year but not currently using an effective method of contraception. These criteria were chosen so that we could assess barriers to contraceptive use generally and specific to COVID-19 among community members at risk for unintended pregnancy and wanting to delay pregnancy, but not using a contraceptive method following the 3-month lockdown. Potentially eligible individuals recommended and introduced to study staff by the VHT completed a brief screening assessment to confirm eligibility with two research assistants. Research assistants obtained written informed consent from eligible and interested participants.

Two facilitators experienced in qualitative research and trained in this study’s focus group protocol moderated the groups, with one leading facilitation and the other taking detailed field notes. The interview guide explored barriers and facilitators to contraceptive use generally and included questions specific to the effect of COVID-19 on pregnancy intentions, risk of unintended pregnancy, access to family planning services, and the ability to use contraception. It also elicited participants’ recommendations for ways to improve family planning delivery during future COVID-19 or other outbreaks/lockdowns. The facilitator conducted the focus groups in Luganda, the local language. The group sessions lasted approximately 90 min each and were audio recorded. Participants received light refreshments during the sessions, and 7,000 Ugandan Shillings (~ $2 USD) for their participation. The audio recordings of FGDs were translated from Luganda to English, translated verbatim, and reviewed by a second party.

In addition, we conducted 15 KIIs with community leaders and family planning stakeholders. Key informants were identified in collaboration with the District Health Team, and recommendations from other local health stakeholders, such as VHTs and the HCIII In-Charges. We sought individuals from the community that could provide expert insight into the local health system, community norms, and other barriers or facilitators to contraceptive use. Thus, KII participants included midwives and other health workers involved in the provision of family planning, VHTs, and community leaders, including religious and elected leaders. We selected KII participants from all three villages and the respective HCIIIs. A trained interviewer conducted one-on-one, in-person interviews in English. Like the FGD guide, the interview guide aimed to elicit determinants of contraceptive use, the effects of COVID-19 on contraceptive use, and recommendations to improve service delivery, but questions were tailored for relevance for each KII. Individuals received 15,000 Ugandan Shillings (~ 4 USD) for their participation. Audio recordings were transcribed and double-checked by a second party. The data that support the findings of this study are available from the corresponding author upon reasonable request.

### Data analysis approach

Data were analyzed thematically [40]. Study transcripts were first reviewed and discussed by study investigators (SK, CM, KS). Based on the initial review of the data, the ABM was selected as a framework to guide the development of a coding scheme to organize barriers and facilitators to contraceptive use in the context of COVID-19. Two trained coders (TH, AO) used an iterative process to apply codes to transcripts. Coders met weekly with KS to discuss new codes and potential themes, and to resolve discrepancies through discussion and consensus. The coders independently coded the transcripts deductively following the coding scheme informed by the ABM framework constructs (e.g., individual predisposing, contextual predisposing), while also applying a code for whether the narrative was COVID-19 specific. New codes drawn inductively from the data were created at this stage. KS reviewed all excerpts after data were fully coded for consensus or re-coding. Codes that represented thematic elements within the ABM framework constructs were collated and a final round of coding was conducted to confirm thematic validity.

## Results

Table 1 displays population characteristics of the focus group sample, which included 13 women and 13 men. On average, the FGD sample was 32 years of age (standard deviation [SD] = 10.36), majority Muslim (69%), with an average of approximately 6 children (SD = 4.09), and most were in polygamous marriages. Women reported greater experience with contraceptives than men; 69% of women had ever used contraceptives within a relationship in their lifetime compared to 15% of men.

**Table 1:** Participant characteristics, focus group discussion participants, Uganda, 2020

	Total (N = 26) n (%)/Mean (SD)	Women (n = 13) n (%)/Mean (SD)	Men (n = 13) n (%)/Mean (SD)
Age	32.27 (10.4)	32.54 (9.8)	32.00 (11.3)
Tribe			
Muganda	24 (92.3%)	13 (100.0%)	11 (84.6%)
Munyarwanda	2 (7.7%)	0 (0.00%)	2 (15.4%)
Religion			
Muslim	18 (69.2%)	10 (76.9%)	8 (61.5%)
Catholic	4 (15.4%)	1 (7.7%)	3 (23.1%)
Protestant	4 (15.4%)	2 (15.4%)	2 (15.4%)
Education			
No grade	4 (15.4%)	2 (15.4%)	2 (15.4%)
Primary	15 (57.7%)	7 (53.8%)	8 (61.5%)
Secondary	6 (23.1%)	3 (23.1%)	3 (23.1%)
Tertiary	1 (3.8%)	1 (7.7%)	0 (0.0%)
Years married	13.46 (10.1)	14.31 (9.78)	12.62 (10.8)
Number of living children	5.73 (4.1)	5.15 (3.7)	6.31 (4.5)
Number of wives			
1	10 (38.5%)	4 (30.8%)	6 (46.2%)
2	13 (50.0%)	7 (53.8%)	6 (46.2%)
3	3 (11.5%)	2 (15.4%)	1 (7.7%)
Ever used family planning			
Yes	11 (42.3%)	9 (69.2%)	2 (15.4%)
No	15 (57.7%)	4 (30.8%)	11 (84.6%)

The 15 KII participants are listed in Table 2 by occupational category and gender. Participants included six health workers (i.e., facility in-charges, family planning focal persons), three VHTs, and six community leaders (i.e., elected political local leaders and religious leaders).

**Table 2:** Key Informant Interview participants by village, Uganda 2020, N = 15

Community position	Gender
Village 1	
HCIII In-Charge/Clinical Officer	Male
HCIII Family Planning Focal Person	Female
VHT Coordinator	Male
Local Council Chairperson	Male
Local Vice Chairperson	Male
Village 2	
HCIII In-Charge/Clinical Officer	Male
HCIII Family Planning Focal Person	Female
VHT	Male
Local Council Representative	Female
Muslim Community Leader	Male
Village 3	
HCIII In-Charge/Clinical Officer	Male
HCIII Family Planning Focal Person	Female
VHT	Female
Local Council Representative	Female
Muslim Community Leader	Male

*HCIII* Health Centre III, *VHT* Village Health Team; local council chairpersons and representatives are elected political officials

**Fig. 2:**
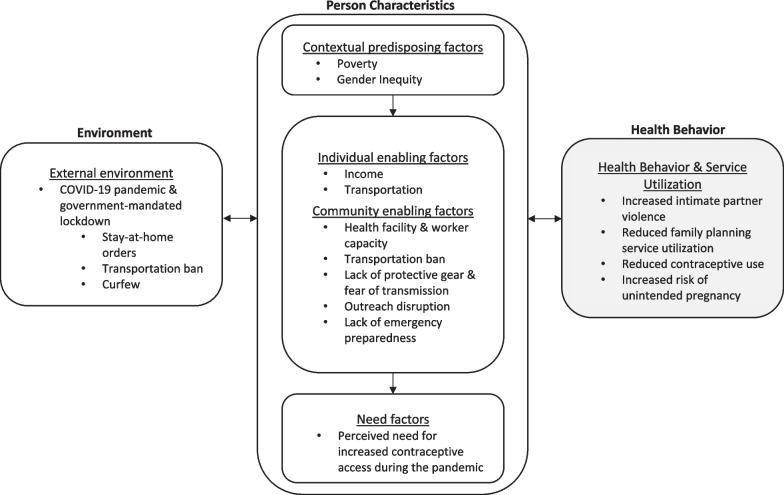
Study findings mapped onto the Anderson Behavioral Model of Health Service Utilization

Study participants described significant disruption to the delivery of family planning services and barriers to accessing health services due to COVID-19. These effects were mainly related to the ramifications of COVID-19 lockdown or prevention measures, as opposed to direct effects of COVID-19. The COVID-19 lockdown measures had effects on individual (e.g., income, transportation) and community enabling factors (e.g., reduced health facility hours, outreach interruptions). Health workers and community members confirmed COVID-19’s direct effect on health service utilization among the community due to fear of infection. Contextual predisposing factors, including poverty and those related to gender inequity, worsened the effect of COVID-19 lockdown on barriers to contraceptive use. Taken together, the data supports COVID-19 prevention measure’s effect on barriers to contraceptive use and other factors that might increase the risk of unintended pregnancy (e.g., increased frequency of sex due to men staying at home). These findings are detailed below, and depicted in Fig. 2, mapped onto the ABM.

### Predisposing factors

#### Contextual predisposing

COVID-19 lockdown restrictions exacerbated existing contextual predisposing factors related to *poverty* and *gender inequity*. For example, the broader context of community poverty exacerbated the effect of COVID-19 on the enabling factor of income (discussed in the next section). As one participant in the women’s focus group (age 36) explained, existing problems related to poverty were made worse by COVID-19:

Respondent: Where [we] operate from to earn [is] locked up. Even if you want to go to the village, it will not work; COVID disorganized everything. Free land is not there anymore, poultry was attacked by different diseases especially the local ones, which we used to operate as free range. The goats were also attacked by several diseases. There is no place where someone can say they can work from or deal in and get some money. All things are disorganized.

Moderator: Have all these things come because of COVID 19 or…?

Respondent: No, they have been there but then COVID has just worsened it all.

In addition, the COVID-19 lockdown intersected with barriers to family planning underpinned by *gender inequity*. Men’s opposition to family planning, reinforced by numerous gender norms linking men’s status to large family size, was one of the most salient barriers to women’s contraceptive use irrespective of COVID-19. As explained by a council representative, “*The opposition [to family planning] – I see it on men’s side. The men are not seriously in support of it. Actually, [the] majority of men strongly disagree with their spouses on the matter of family planning, both Muslims and Christian*” (Local council representative, female). Power imbalances between men and women, an outcome of inequitable gender norms, result in male control of family planning decision-making. Consequently, all participants agreed that it is common practice for women in the community to use family planning in secret, but health workers explained how COVID-19 stay-at-home orders disrupted women’s ability to do so. The following quotation from a female healthcare worker demonstrates how stay-at-home orders have reduced women’s autonomy to use contraceptives:

“A woman will come and tell you that, ‘Musawo [Doctor], please hurry up because I lied to my husband that I had just gone to the neighbor’s place.’ So when you hear that, you leave whatever you are doing and decide to go and attend to this mother. So, this lockdown has really affected them because the man is always around and he is so inquisitive. When they go to the health facility they are asked why they have delayed, he [man] even times her. He can say you went in the morning, how come you have spent all that time?”

Also related to gender inequity, while participants generally looked down on IPV, they agreed that it was still prevalent in the community, with some male participants considering it necessary in some situations. The stay-at-home orders were said to have resulted in an increase in IPV in general: “*COVID-19 has mainly brought violence in homes*” (women’s focus group, age 21). Further, IPV was an expected response to finding one’s wife using family planning in secret, thus potentially diminishing women’s use during COVID-19, as one woman explained: “*Physical abuse is not supported in the community, but if it concerns family planning, most men support the abuse*” (women’s focus group, age 23).

Gender inequity also intersected with the COVID-19 pandemic to increase women’s risk of unintended pregnancy through the increased occurrence of sex due to men being at home more; see excerpt below from the women’s FGD.

Respondent 1 (age 42): Most men are at home; they lack what to do and they are all over their wives all the time and [the] majority of women are pregnant now.

Respondent 2 (age 36): I have nothing different [to say] but we are bored of men being around us all the time.

Moderator: Okay, but what has led to these women getting pregnant during the COVID lockdown?

Respondent 1: We used to count our days [calendar method] and things would be okay but today they [men] are around us all the time. You would spend your 10 days or more without being disturbed but today they [men] are home all the time.

This and other narratives imply that women may not be interested in having more sex, but due to inequitable gender norms, have less say over its occurrence than men in a relationship.

### Enabling factors

#### Individual enabling

COVID-19 had a negative effect on *income*, an individual enabling factor, in turn affecting service access. As one female focus group participant (age 23) stated, “*For me, this lock down has affected me in my wellbeing and income; all [of] my business has gone down. We are so poor in this lock down*.” Participants explained how the pandemic’s effect on income created new financial barriers to accessing health services, particularly in not having money to cover the cost of *transportation* to the clinic, another enabling factor, or the cost of methods at private clinics, as the following exchange between participants from the younger women’s focus group illustrates:

Participant 1 (age 24): Now the method can be due but when you don’t have money for transport to go to the health centre and get the new dose or even for the injectaplan. So, the next thing is to conceive because you are married but the family planning method expired.

Participant 2 (age 23): Also, such women may get money for transport and may fail to get money for the injectaplan [at private clinics] or fail to get transport but remember they escape from home to go for it to a health centre. So, you cannot walk; you will be delayed – bearing in mind that you escaped from home, you need to run and come back soon.

This narrative again intersects with the contextual predisposing factor of *gender*; women that use family planning in secret tend to have more difficulty getting to the clinic.

#### Community enabling factors

Community enabling factors were prominent barriers to contraceptive use pre-dating COVID-19, including contraceptive method and equipment stock outs, low provider capacity to provider LARCs such as IUDs at HCIIIs, clinic wait time, and the perception of poor treatment from providers. Facilitators, irrespective of COVID-19, included community outreach strategies, such as VHT outreach and collaboration with Marie Stopes International for pop-up clinics. COVID-19 worsened existing and created new barriers to family planning within community enabling factors. For example, during lockdown, family planning services had *lower capacity* due to fewer health workers available and shorter hours. Moreover, a common misconception in the community was that facilities were closed completely, resulting in poor attendance, as one female health worker explained: *“New [contraceptive] users were even more affected. They thought that family planning services were also locked-down. It's mainly after a period of about two months that we started to see some of the new users*.” Moreover, health worker participants described *fear of COVID-19 transmission*, as well as a *lack of protective gear*, as a barrier to the delivery and receipt of services. As one female health worker explained, “*It [COVID-19] has really affected me because most of my work has been door-to-door, so it worries me to move to people’s homes because I can infect them or they can infect me.*" Health workers described the risks of being without protective masks, gloves, thermometers, and hand sanitizer throughout the 3-month lockdown. As another male VHT explained, this also negatively affected client attendance: *“The family planning clients fear to meet health workers and *vice versa* because they don’t have masks.”*

COVID-19 lockdown’s effect on mobility was the most salient community enabling barrier to family planning delivery. Lockdown included a *strict transportation ban* that restricted all public transportation (i.e., taxis, boda bodas or motorcycle taxis), which negatively affected the community’s access to health facilities; those that do not live in walking distance rely on public transportation to get to the clinics. Some transportation exceptions were made for “essential services,” such as for pregnant women reaching the clinic. Although the District provided transportation for healthcare workers to reach the facilities, the transportation ban still challenged the movement of healthcare workers, making it more difficult to get to the facilities for work, and prohibited the distribution of contraceptives and family planning education through community *outreach* from Marie Stopes International and VHTs. The community relies on quarterly outreach from the non-governmental organization, Marie Stopes International, to provide contraceptives not offered at the local clinic because of limited provider capacity to offer LARCs and frequent stock outs. This outreach was restricted during lockdown.

“Between March and June, we stopped the outreaches because there was a total ban on gatherings. In that quarter, we never held any outreaches and the outreach is our biggest clinic because people know that is when Marie Stopes, that has the experience, is coming. So, in that quarter the uptake was low, people were not coming out and they also had their own challenges with transportation to the facility” (Healthcare worker, male).

In addition, as explained by one male VHT, the movement of VHTs was restricted during lockdown: *“The lock down affected our mobilization and counseling because of the rules of social distancing. We could not access community members.”* VHTs who normally mobilize the community, providing individual and group outreach, education, and short-term contraceptives, were prohibited from working in the community.

“As health workers, we remained open but working for fewer hours than full days. Of course, we kept receiving complaints from the community about the inaccessibility of the patients who wanted us to help them, but we could not help; we had no means to reach out to help them. The VHTs would bring complaints from the community expecting quick feedback on how we could reach out to communities but we had no means to do it. Besides, we had to adhere to the guidelines from the government” (Healthcare worker, female).

As the above quotation explains, there was *a lack of emergency preparedness*, with no protocols in place to ensure client needs could be met during the pandemic, and government restrictions did not include an exception for outreach of family planning services.

### Need

Health workers, VHTs, and community leaders recognized a high need for family planning in the community based on a shared perception that contraceptive use in the community was low alongside high fertility and limited resources to support large families. While women endorsed family planning much more than men, both women and men perceived a need for family planning from a socioeconomic standpoint; there was consensus that families should only have the number of children they can afford to care for. In addition, women discussed the health and social benefits of spacing their children. While participants recognized that COVID-19 lockdown measures had a negative effect on access to contraceptives, and thus, a need for increased access to services during the pandemic, they did not express any new need for or reasons for wanting contraceptives related to COVID-19 itself. That is, women and men in the FGDs did not say COVID-19 changed their pregnancy intentions or the spacing and timing of their future pregnancies.

### Perceived effect on health outcomes

As described above, participants perceived that COVID-19’s effects on predisposing and enabling factors likely resulted in lower contraceptive use through restrictions that affected health service delivery and access. As such, risk of unintended pregnancy was thought to have increased due to a lack of access to services, but also through the increased occurrence of sex. Further, participants perceived a rise in IPV at home due to COVID-19, which could further diminish women’s autonomy of contraceptive use and ability to refuse sex with one’s husband, also contributing to risk of unintended pregnancy.

### Recommendations for family planning service delivery in the context of COVID-19

Participants’ recommendations to improve contraceptive use in the context of COVID-19 centered on strengthening emergency preparedness and response. *“There should be prior arrangements to equip health facilities by [the] government with other stakeholders, with means of transport like motorbikes, vehicles, and the government should train staff for outreaches in case of similar eventualities as this one [COVID-19 lockdown].”* As this quotation from a female health worker exemplifies, recommendations included making special provisions for transportation that would allow family planning users to continue to access services through public transport in the event of a COVID-19 transportation ban, as well as strengthening community-based family planning (CBFP). Participants suggested adapting existing CBFP approaches so that they could be safely implemented during a COVID-19 outbreak, including continued distribution through Marie Stopes International and VHTs. Health workers, VHTs, and community members were in support of building VHT capacity to deliver methods, such as injectable contraceptives irrespective of COVID-19, and noted that it would be particularly beneficial during a COVID-19 outbreak. An external project had previously trained some VHTs in the district in injectable contraceptives, but implementation became inconsistent after the project ended.

Recommendations for new CBFP approaches to ensure contraceptive access amid COVID-19 included the creation of a voucher system that would link governmental facilities to private shops/pharmacies, allowing people to access contraceptives from local shops for free. Participants explained that bringing free methods to nearby shops would reduce barriers to access during a COVID-19 outbreak, including transportation barriers and partner disapproval, as one could access the private shops more discreetly. Participants also recommended mobile services, such as pop-up tent clinics or delivering methods as part of community dialogues, which would simultaneously generate demand for family planning. Participants discussed how such venues could be set up to incorporate COVID-19 prevention protocols in an outdoor or semi-outdoor space, allowing for ventilation and social distancing. While VHT and private shop distribution were viewed favorably by women and health workers because they could allow women to access contraceptives without their partners’ knowledge, FDGs and KII narratives made clear that issues of gender inequity and male control over contraceptives need to be considered when planning more public outreach events. Specifically, engaging and sensitizing men was viewed as essential to the success of such efforts, as the following quotation from a male VHT demonstrates:

“The community dialogues, in my view, can easily create hostilities and conflicts in homes. Remember that in our community men discourage family planning use because of religion and culture. The men will have to also attend those dialogues, or someone would tell them what transpired in those meetings. I see that this kind of strategy would not be effective, unless you first provide counseling and education [for] men separately and women separately and make sure that their spouses are in agreement.”

Finally, health workers emphasized the need for protective gear (e.g., masks, gloves, hand-sanitizer) and COVID-19-related health worker training. Training was requested to ensure methods could be delivered while minimizing risk of transmission, but health workers recognized the need for training to also reduce fear and panic, as a male health worker stated, “*We need psychosocial counseling [for] health workers because everyone was in panic saying, ‘I might infect my family.’ The In-Charge had to explain to the people all the time that they had to work.*” Pointing to health system weaknesses that pre-date the pandemic, providers also recognized the need for capacity building and refresher trainings on family planning services generally, as the following exchange between a male health worker and the moderator exemplifies:

Respondent: I think training is key, because if we are aware that indeed the disease is spread, and if you have the protective equipment, I think that gap can be reduced.

Moderator: So, you are saying sensitizing health workers or educating them about the outbreak and equipping them?

Respondent: Yes, and also providing confidence to them [health workers].

Moderator: What else?

Respondent: Even training on the skills, for example, in general; it may not be for COVID-19 but like this refresher training, like in general, inserting IUDs and also training us on managing side effects.

## Discussion

This qualitative study used the Andersen Behavioral Model of Health Services (ABM) to understand the effects of COVID-19 on family planning barriers and facilitators in a rural area of Uganda. Participants described significant disruptions to the delivery of family planning services, and access to health services generally, due to COVID-19 during and after Uganda’s first government-mandated COVID-19 lockdown in 2020. COVID-19 had direct effects on enabling factors at the individual-level, such as reduced income negatively affecting individual’s access to transportation and private services, but the larger consequences were at the community-level, with the suspension of CBFP being particularly disruptive. In addition, COVID-19 exacerbated contextual predisposing factors that served as barriers to family planning before the pandemic, including poverty and those related to gender inequity, such as IPV and power inequities between men and women that diminish women’s ability to refuse sex with their husband and their autonomy to use contraceptives. Recommendations to improve family planning service delivery in the context of COVID-19 centered on emergency preparedness and strengthening existing community distribution and creating new ways to safely deliver contraceptive methods directly to communities during future COVID-19 lockdowns. Although this qualitative study is not a direct measure of COVID-19’s effect on contraceptive use or unintended pregnancy, this study highlights potential pathways in which COVID-19 may directly (e.g., through increased fear of going to the clinic) and indirectly (e.g., through lockdown) result in unintended pregnancies via reduced contraceptive use, increased sex from men being at home, and increased IPV.

While a number of commentaries and modeling studies exist that project the effects of COVID-19 on women’s reproductive health outcome [1, 9–11], few studies to-date have used primary data collection to explore the effect of COVID-19 on family planning barriers in LMICs. Those that have primarily use cross-sectional surveys, which report reduced access to family planning and related services during the pandemic [12–15]. One Ugandan study reported 31% of respondents were not able to use a LARC during lockdown, while a South African study reported 22% of their sample could not access condoms. A longitudinal study in Burkina Faso and Kenya reported the majority of women at risk of unintended pregnancy did not change their contraceptive status during COVID-19; however, approximately 14% of non-contraceptive users in Kenya and 4% in Burkina Faso identified COVID-19-related reasons for non-use [16].

To our knowledge, the present study is one of the first qualitative studies to examine the potential effects of COVID-19 on family planning in a LMIC. Thus, this study adds context to the limited quantitative research that exists by illuminating different ways in which COVID-19 and its consequential lockdown and prevention measures have affected family planning barriers and exacerbated existing gender disparities and health system weaknesses. Narratives from our community sample described COVID-19’s disruption to community resources that enable access to family planning services (i.e., community enabling factors). This included reduced facility capacity and community outreach disruptions, as researchers predicted early in the pandemic [1] and other have since reported [13, 14, 41]. Participants cited Uganda’s transportation ban as a major barrier to accessing care; transportation remained a barrier to care post lockdown due to COVID-19’s negative effect on the individual enabling factor of income. Sseninde and colleagues [14] reported similar individual and community enabling factors affected by COVID-19 during Uganda’s lockdown, including stock out of contraceptive methods, a lack of transportation, and a lack of money to access care. In the present study, the slow provision of masks, thermometers, and other protective supplies from the government resulted in widespread fear of social interaction, which reduced client willingness to come to the clinic and created fear among clients and health workers.

These findings point to a lack of emergency preparedness that would allow for the continued and safe provision of family planning services amid an infectious disease outbreak or other humanitarian crises. Participants’ recommendations highlighted the need for COVID-19 training and capacity building, access to protective supplies, and client access/transportation to the facility by deeming family planning an essential service. In addition, community members and health workers alike in our sample recommended the strengthening of CBFP to bring services safely to communities. Mobile services, VHTs (or community health workers [CHWs]), and distribution of methods through local pharmacies and shops were strategies recommended by our sample to deliver family planning while limiting facility-based contact and reducing barriers to care resulting from travel restrictions. These methods are considered High Impact Practices (HIPs), or evidence-based strategies for family planning [42], and have been recommended by others as strategies to overcome COVID-19 related challenges to service delivery [11, 43–45]. The present study also highlights the need for general health system strengthening to address issues predating the pandemic, such as stock challenges and low capacity for LARC provision. Lessons learned from previous infectious disease outbreaks, such as the 2013 Ebola epidemic in West Africa, can inform health systems resilience-building and the delivery of essential services amid public health or other humanitarian crises [46–48].

Our findings suggest that the success of CBFP during a public health or other humanitarian crisis, however, would be contingent on simultaneous efforts to increase male support for family planning. In this rural community, men largely opposed family planning and had a strong influence over women’s autonomy to use contraceptives. CBFP would make contraceptive use more public, reducing women’s ability to use contraceptives in secret. These findings align with a large literature demonstrating the need to increase male support of family planning and women’s use of reproductive healthcare generally in order to increase service uptake in LMICs [49–51]. Participants in our sample perceived COVID-19 stay-at-home orders to have compromised women’s ability to use family planning in secret, while further increasing women’s risk for unintended pregnancy through increased sex, which women may have less say over than men in a relationship. Moreover, our sample perceived COVID-19 to have resulted in an increase in IPV, and that IPV would be a generally accepted response to finding a spouse using family planning in secret. In a review of 38 studies, Sánchez et al. [52] reported that factors that increase women's vulnerabilities to violence were exacerbated during COVID-19’s social distancing and lockdown period. Researchers have similarly speculated that the pandemic would have more negative effects on women through the deepening of existing gender inequities [18, 53]. Our study contributes to this literature by illustrating the ways in which the pandemic worsened the contextual predisposing factor of gender inequity on contraceptive use and risk of unintended pregnancy.

These findings point to the need to address the underlying gender inequities that already impede women’s use of contraceptives in rural settings. Interventions to engage men in family planning or sexual and reproductive health services have had mixed success [54–57]. However, tailored messaging to men’s interests, improving partner communication and gender equitable attitudes, and demonstrating family planning support from other men are intervention strategies that show promise [55, 58–63]. More research is needed on strategies that can be implemented alongside CBFP to increase male support for family planning and generate demand for family planning generally. Participants in our sample suggested community dialogues to increase the acceptability of CBFP. A community dialogue intervention in Kenya focused on gender equity and family planning increased family planning acceptability among participants and resulted in increased contraceptive use among women [64].

## Limitations

Readers should interpret the findings of the present study with the study’s limitations in mind. While this study can shed light on the effects of COVID-19 on family planning barriers and facilitators in rural LMIC settings, the findings may not be generalizable to other dissimilar settings. This qualitative study explores the perceived effects of COVID-19 on family planning outcomes but is not a direct measure of these outcomes. For our focus groups, we sampled men and women of reproductive age with an unmet need for family planning (i.e., they reported wanting to delay their next pregnancy but were not using an effective method of contraception). Thus, these participants were able to share their reasons for non-use, including those related to COVID-19, but do not provide insight into barriers to continued use of contraceptives among current users.

## Conclusion

As the COVID-19 pandemic continues to devastate LMICs globally, and inequities in global vaccine distribution continue to favor high-income countries [65], efforts must be made to ensure the safe distribution of health services amid ongoing transmission surges and subsequent lockdowns. The present study highlights the negative impact COVID-19 had on family planning barriers in a rural area of Uganda post-COVID-19 lockdown. This study highlights the need for health system strengthening in general, and emergency preparedness specific to family planning and COVID-19. Moreover, we join others in calling for family planning services to be included as an essential service during lockdown or other public health crises that disrupt regular service provision [12, 14]. Community-based distribution of family planning in particular should be explored, but will not be successful unless efforts are made to simultaneously address existing relationship-level barriers to contraceptive use. This study highlighted prevalent cultural norms that reinforce gender inequity as barriers to women’s autonomous use of contraceptives, which were exacerbated by COVID-19. The lessons learned from this study can be used to strengthen health and community systems against future COVID-19 outbreaks, but can also be applied to public health and humanitarian crises more broadly.

## Data Availability

The datasets analyzed during the current study available from the corresponding author on reasonable request.

## Abbreviations

ABM: Andersen’s Behavioral Model of Health Service Use
CBFP: Community-based family planning
CHWs: Community health workers
FGD: Focus group discussions
HCIII: Health center III
HIP: High Impact Practice
HCV: Health center V or a general hospital
IUD: Intrauterine device
IPV: Intimate partner violence
KII: Key Informant Interviews
LARCs: Long-term reversible methods (LARCs)
LMICs: Low and middle-income countries
SD: Standard deviation
UNFPA: United Nations Population Fund
USD: United States Dollar
VHT: Village Health Team
